# An investigation on conjugated linoleic acid content, fatty acid composition, and physicochemical characteristics of Iranian Kurdish butter oil

**DOI:** 10.1002/fsn3.3142

**Published:** 2022-12-07

**Authors:** Rezgar Faraji Sarabmirza, Parham Joolaei Ahranjani, Ladan Rashidi, Mohammad Mousavi, Faramarz Khodaiyan, Hamid Rashidi Nodeh

**Affiliations:** ^1^ Department of Food Science and Technology, Campus of Agricultural Engineering & Natural Resources University of Tehran Karaj Iran; ^2^ Department of Microbial and Molecular Systems, Faculty of Bioscience Engineering KU Leuven Leuven Belgium; ^3^ Food Technology and Agricultural products Research Center Standard Research Institute (SRI) Karaj Iran

**Keywords:** conjugated linoleic acid, fatty acid composition, Iranian Kurdish butter oil, Kurdish Roghan, physiochemical characteristics

## Abstract

In this study, physicochemical and quality properties, fatty acid composition, and triglyceride composition of Iranian Kurdish butter oil (IKBO) obtained from yogurt drink (doogh) butter were investigated. Local doogh butter, prepared from cow's (CIKBO) and ewe's milk (EIKBO), was utilized as the raw material for this purpose. The free fatty acids (FFA) and peroxide values of IKBOs of the cow (CIKBO) and ewe (EIKBO) were obtained at 0.41 ± 0.01 and 0.39 ± 0.01 (g Oleic acid 100/g oil), and 1.32 ± 0.00 and 1.35 ± 0.00 (meq O_2_ kg/oil), respectively. The amounts of saturated fatty acids (SFAs): 70.27 ± 0.62 and 72.13 ± 0.84 (g/100 g), monounsaturated fatty acids (MUFAs): 19.37 ± 0.74 and 20.56 ± 0.97 (g/100 g), and polyunsaturated fatty acids (PUFAs): 1.22 ± 0.12 and 2.75 ± 0.38 (g/100 g) were obtained in CIKBO and EIKBO, respectively. The significant majority of the fatty acids (FAs) in the examined CIKBO and EIKBO were myristic (CIKBO: 13.76 ± 0.02 (g/100 g) and EIKBO: 14.83 ± 0.07 (g/100 g)), palmitic (CIKBO: 33.14 ± 0.28 (g 100/g) and EIKBO: 31.86 ± 0.02 (g/100 g)), stearic (CIKBO: 8.27 ± 0.06 (g/100 g) and EIKBO: 7.95 ± 0.06 (g/100 g)), capric (CIKBO: 4.83 ± 0.03 (g/100 g) and EIKBO: 6.75 ± 0.01 (g/100 g)), and oleic acids (CIKBO: 15.37 ± 0.12 (g/100 g) and EIKBO: 17.83 ± 0.02 (g/100 g)). The average of conjugated linoleic acid (CLA) content in EIKBO (2.20 ± 0.22 (g/100 g)) was higher than that in CIKBO (0.92 ± 0.25 (g/100 g)) (*p* < .05). Therefore, EKIBO is considered the superior natural supply of CLA.

## Graphical abstract

Local doogh butter was prepared from cow's (CIKBO) and ewe's milk (EIKBO). Then, the physicochemical properties, fatty acid composition, and triglyceride composition of CIKBO and EIKBO butter were investigated with gas chromatography (GC‐FID). Therefore, the GC‐FID profile is provided a superior natural supply of CLA and low molecular weight fatty acids.

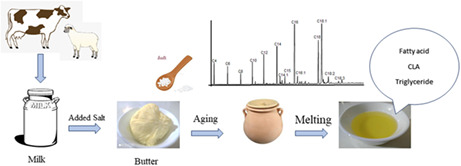

## 1 INTRODUCTION

Since the Achaemenid era, Iranian Kurdish butter oil (IKBO) has been manufactured from doogh butter. It is salted and allowed to ripen for 2–6 months before being heated to separate the fat milk from the serum milk (Batmanglij, 1999). Locally known as “Kurdish Roghan,” it is a frequently consumed item in Iran's northwestern provinces. Butter is traditionally made by churning doogh, which is made from goat, ewe, or cow milk (Batmanglij, 1999). Numerous studies have examined the fatty acid content and composition of butter oil, butter, and milk fat (Collomb et al., 2002; Glew et al., 1999; Sağdıç et al., 2004); however, there is no publication on the properties of IKBO (Kurdish Roghan).

Due to their high saturated fatty acids (SFAs) content, dietary milk fats have always been related to arteriosclerosis and coronary heart disease (Grundy, 1997; Lokuruka, 2007). Nonetheless, current research has concentrated on the beneficial components of milk lipids, including conjugated linoleic acid (CLA) (Crumb & Vattem, 2011; Nagpal et al., 2012). The nutritional benefits of CLA consumption have been demonstrated in vitro and animal studies. These benefits include anticarcinogenic characteristics (Parodi, 1997), antiadipogenic characteristics (House et al., 2005), reduction of LDL cholesterol and heart disease (Crumb & Vattem, 2011), and lean body mass enhancer (Park et al., 1997). CLA refers to a blend of linoleic acid's conjugated positional and geometric isomers (cis‐9, cis‐12 octadecadienoic acid). Milk fat is the most remarkable natural source of CLA since it contains cis‐9, trans‐11‐18:2, also known as rumenic acid (RA), accounting for more than 90% of the CLA isomers present (Bauman & Griinari, 2003; Parodi, 2003). Rumenic acid (cis‐9, trans‐11 CLA) is produced in ruminants as the first intermediate as a result of the incomplete biohydrogenation of polyunsaturated fatty acids (PUFAs), particularly linoleic acid (LA) and α‐linolenic acid (LNA), by Butyrivibrio fibrisolvens in the rumen (Kepler & Tove, 1967; Khodamoradi et al., 2013), as well as the action of ∆9–desaturase enzyme on vaccenic acid (C18:1 trans‐11) to create rumenic acid inside mammary glands tissues (Griinari et al., 2000).

Butter and ghee are the largest sources of cis‐9 and trans‐11‐octadecadienoic acid, and it has been claimed that their intake may alter CLA levels in blood serum and human milk (Parodi, 1994; Sindhuja et al., 2020). Through the biohydrogenation pathway's isomerase and reductase enzymes, microbial fermentation leads to an increase in CLA content during ghee production (Aneja & Murthi, 1990; Mehta, 2009).

The milk's fatty acid content is critical for processing and human health (Kay et al., 2005). Some studies are being conducted in several countries on the CLA content and the composition of FAs in dairy products (Donmez et al., 2005; Guler et al., 2010; Indu & Jayaprakasha, 2021; Kelly et al., 1998; Ledoux et al., 2005). The CLA content and the composition of FAs of IKBO are unknown. Therefore, this study aimed to evaluate the physicochemical and quality properties, fatty acid composition, and triglyceride composition of Iranian Kurdish butter oil (IKBO) (the traditional and beneficial oil) obtained from yogurt drink butter named doogh, which is prepared from cow's and ewe's milk (as the raw material).

## 2 MATERIALS AND METHODS

### 2.1 Materials

#### 2.1.1 Raw milk

Fresh raw milk (cow and ewe) was sourced in Kurdistan, Iran's northwest region. The physicochemical quality properties of raw milk were determined according to the described methods by the International Organization for Standardization (ISO), including titratable acidity (ISO 660, 2020), dry matter (Uzatici & Yayintaş, 2017), fat content (ISO 19662, 2018), and pH (ISO 26323, 2009). Yogurt was applied as the starting culture produced in the laboratory. All studies were conducted at the dairy pilot plant in the department of food science and technology of the University of Tehran, Iran.

### 2.2 Methods

#### 2.2.1 Butter oil production

To get butter oil samples, the following technique was performed. Raw whole milk (cow and ewe) was utilized to make yogurt without additional processing. The fresh milk was heated to 90°C for 15 min, inoculated with the starting culture, and incubated at 40–42°C until the pH reached 4.6–4.7, at a certain point, then cooled to 4–5°C to end the fermentation. Next, the yogurt samples were kept at 4–5°C for 12–14 h to crystallize the milk fat. Subsequently, enough hot water (1:1 vol) was added to obtain the desired doogh (Iranian yogurt drink) and churned at 14–16°C for about 30 min by a pilot churning machine (Model 2585 Crealan Package (CP), Chicago). The buttermilk was removed after the churning process, and the butter grains were rinsed twice with cold water (7–9°C) to eliminate any remaining buttermilk. Fresh butter samples (ewe and cow) were put in glazed pots and rubbed with an appropriate quantity of dry salt (10% w/w) before being kept at 18 ± 2°C for 4 days. The samples were returned multiple times over this period. After this first interval, dry salt was applied to cover the butter samples thoroughly, forming a continuous salt coating throughout the whole butter surface. The pots were then carefully covered and kept at 18 ± 2°C for 4 months to mature. The samples were then melted at 90 °C and separated into oil and serum phases. At 110°C, the melted butter samples were swirled until the oil became a light brown hue. The oil phase was clarified by filtering it through multiple layers of cheesecloth and storing it at −24°C until further analysis. Two distinct sets were prepared for analysis using the technique outlined above.

#### 2.2.2 Physicochemical properties of butter oil

The fat content of obtained butter oil was measured using Internationaler (1966); Codex Standard (1999); amended 2018. Briefly, butter oil samples were warmed and melted in a water bath at 40–50°C till the water separated. After 5 min of centrifugation at 3500 rpm, the transparent supernatant was filtered using filter paper.

pH values were obtained using a pH meter (GLP 22, Crison, European Economic Community). Additional physicochemical characteristics, including saponification value, iodine value, moisture, melting point, and refractive index of butter oil samples, were determined according to the described methods by the Association of Official Agricultural Chemists (AOAC) (AOAC's scientific standards and methods, 2019).

##### Determination of the peroxide value

The peroxide values of the obtained fat from butter oil samples were determined according to the described method in ISO 3976 (2006).

##### Determination of the free fatty acids (FFAs)

The FFAs’ values of the obtained fat of butter oil samples were measured according to ISO 660 (2020).

#### 2.2.3 Determination of fatty acid composition

The butter oil samples were immediately warmed at 45°C and vortexed. Fatty acid methyl esters (FAMEs) of butter oil samples were prepared according to the described method by the ISO 15884 (2002) and analyzed using a GC Varian 3400 (USA) equipped with a flame ionization detector (FID) and a CP‐SIL‐88 capillary column (100 m × 0.25 mm i.d., film thickness 0.25 μm). Helium as the carrier gas at a pressure of 20 kPa was applied. The injector and detector were adjusted at 280 and 300°C, respectively. The oven was kept at 60°C for 5 min, elevated to 180°C at a rate of 5°C/min, and then accelerated to 190°C at a rate of 2°C/min and kept at this temperature for 25 min. The split ratio was 1:50. The retention time of the observed peaks was compared to those of an authentic standard FAME mixture purchased from Sigma‐Aldrich Company (St. Louis, MO).

#### 2.2.4 Triglycerides (TAGs) composition

Triglycerides (TAGs) were determined according to the specified reference method described in the ISO 17678 (2019) using gas chromatography equipped with a flame ionization detector (FID) (Yung Lin 6100, Korea) with an injector on‐column.

An Agilent CP‐TAP CB, 0.25 mm × 25 m WCOT fused silica coated with TAP (df = 0.1 μm) was applied, and temperatures of the injector and detector were adjusted at 360°C and 340°C, respectively. The temperature of the furnace was started at 200°C, increased at a rate of 5°C/min, and reached 300°C after 30 min, then increased at a rate of 5°C/min and reached 355°C. Hydrogen was applied as the carrier gas at a pressure of 100 kPa. The mixture standard of triglycerides (C24–C54) was purchased from Sigma‐Aldrich (USA). It is necessary to verify that the standard error of the user's repeatability research results meets the guidelines specified in the European Legislation (Commission Regulation (EC) No 454/95, 1995) defining the source procedure for detecting foreign lipids in milk fat using GC assessment of triglycerides. The stock solutions of TAGs were prepared in methanol (1 mg/ml), then working solutions of TAG standards were made by diluting the stock solution. The identification and quantification of the peaks were performed by a mixture of eight standard triglyceride methods (Fontecha et al., 1998).

#### 2.2.5 Statistical analysis

Data analysis was gained at least in triplicates and averaged. One‐way analysis of variance (ANOVA) was used for statistical analysis. *p* ≤ .05 was taken into account as statistically significant. The results are presented as mean ± standard deviation (SD). Excel software (Microsoft, Redmond, WA) was applied for linear regression analysis to check the linearity of the detector response and for calculating average and SD values.

## 3 RESULTS AND DISCUSSION

Table 1 summarizes the qualities of fresh raw milk samples used as the raw material for preparing IKBO. Physicochemical characteristics, including pH, TA, dry matter (DM), and fat contents of raw milk used for the preparation of butter oil, are presented in Table 1.

**TABLE 1:** Physicochemical properties of raw milk^a^.

Parameter	Cow	Ewe
pH	6.70 ± 0.01^a^	6.68 ± 0.01^b^
Titratable acidity (g 100/g)	1.12 ± 0.02^a^	1.45 ± 0.02^b^
Dry matter (g 100/g)	12.45 ± 0.01^a^	18.37 ± 0.02^b^
Fat (g 100/g)	3.75 ± 0.01^a^	7.80 ± 0.01^b^

*Note*: Means in the identical raw with distinct superscript letters differ *p* < .05.

^a^ Mean of three replicates.

These qualities are determined by physiochemical parameters and compliance with sanitary‐hygienic milking requirements. In order to determine the needed features, numerous tests are available (for instance, titratable acidity (TA) is a quick test (90 s to complete) that indicates the quality of raw milk and offers an indirect assessment of the acid level in milk).

According to the obtained results, the fat content in ewe milk is higher than that in cow milk; therefore, ewe milk fatter than cow milk. In addition, the dry matter content and acidity value of ewe milk were higher than those in cow milk.

### 3.1 Physiochemical properties of the Iranian butter oil

Table 2 summarizes the physicochemical characteristics of prepared Iranian butter oil samples. According to the results, the fat contents of butter oil samples were more than 99% (g/100 g), which was comparable to other traditional butter oils. It was found that the physicochemical properties of CIKBO and EIKBO samples were different. According to the obtained results, it can be seen that the EIKBO sample had higher amounts of refractive index, pH, iodine and saponification, and butter oil fat (g/100 g) than the CIKBO sample. The findings indicated that a substantial difference in the saponification and iodine values, moisture content, pH value, fat content, peroxide value, and FFAs value was observed in both CIKBO and EIKBO samples (*p* < .05). Results showed higher fat content, pH value, iodine and saponification values, and refractive index in the EIKBO sample, while the moisture content, melting point value, and FFAs value of the CIKBO sample were higher than those in EIKBO. The peroxide values of the fat samples were much lower than those reported by Fındık and Andiç (2017), ranging from 1.32 to 1.35 (meq O_2_/kg oil) (*p* < .05). Also, the peroxide values of samples were obtained lower than the specified limit of 1.5 (meq O_2_/kg oil) in the Iranian national standard (INSO 1254, 2020). The amounts of free fatty acids (FFAs) were lower than the permitted limit of 0.8 (g Oleic acid 100/g) in the INSO 1254 (2020). The moisture content limitation in INSO 1254 is 0.5 (g/100 g oil), which results in the moisture contents of EIKBO and CIKBO being lower than this value. These findings are consistent with previous reports (Sağdıç et al., 2004; Şenel et al., 2011).

**TABLE 2:** Physicochemical characteristics of Iranian butter oil samples^a^.

Parameters	CIKBO	EIKBO
Moisture (g 100/g)	0.36 ± 0.02^a^	0.12 ± 0.01^b^
Fat (g 100/g)	98.14 ± 0.12^a^	99.38 ± 0.15^b^
pH	4.65 ± 0.05^a^	4.72 ± 0.03^b^
Free fatty acid (g Oleic acid 100/g)	0.41 ± 0.01^a^	0.39 ± 0.01^b^
Iodine value (ml 0.1 N Na_2_S_2_ O_3_/g)	33.38 ± 0.38^a^	35.40 ± 0.25^b^
Saponification value (ml 0.5 N HCl/g)	240.80 ± 0.51^a^	274.12 ± 0.42^b^
Peroxide value (meq O_2_/kg oil)	1.32 ± 0.00^a^	1.35 ± 0.00^b^
Refractive index (35.8°C)	1.4574 ± 0.000^a^	1.4580 ± 0.000^b^
Melting point (°C)	33.78 ± 0.07^a^	30.60 ± 0.09^b^

*Note*: Produced from different mammals. Means in the identical raw with distinct superscript letters differ *p* < .05.

Abbreviations: CIKBO, Iranian Kurdish butter oil produced from cow's milk; EIKBO, Iranian Kurdish butter oil produced from ewe's milk.

^a^ Mean of three replicates.

### 3.2 Fatty acid composition and CLA content of IKBOs

Table 3 represents the fatty acid composition and CLA content of the samples. Twenty‐seven fatty acids were detected and quantified in IKBO samples. Five fatty acids, including capric acid (C10:0), myristic acid (C14:0), palmitic acid (C16:0), stearic acid (C18:0), and oleic acid (C18:1), accounted for more than 70% of total fatty acids in Iranian butter oil samples from cows and ewe. As shown in Table 3, palmitic acid (31.86–33.14 (g 100/g butter oil)), oleic acid (15.37–17.83 (g 100/g butter oil)), myristic acid (13.76–14.83 (g 100/g butter oil)), stearic acid (7.95–8.27 (g 100/g butter oil)), and capric acid (4.83–6.75 (g 100/g butter oil)) had large percentages in the samples, but palmitic acid was the predominant fatty acid. The specified values of palmitic acid, oleic acid, myristic acid, stearic acid, and capric acid in INSO 1254 were 25–41 (g 100/g butter oil), 18–33.4 (g 100/g butter oil), 5.4–14.5 (g 100/g butter oil), 6–15 (g 100/g butter oil) and 1.7–3.9 (g 100/g butter oil), respectively.

**TABLE 3:** Fatty acid composition and CLA content of IKBO samples^a^.

FAMEs	CIKBO (g 100/g butter oil)	EIKBO (g 100/g butter oil)
C4:0	1.16 ± 0.02^a^	0.98 ± 0.03^b^
C5:0	0.25 ± 0.04^a^	0.20 ± 0.06^b^
C6:0	2.64 ± 0.03^a^	2.10 ± 0.08^b^
C8:0	1.28 ± 0.03^a^	2.62 ± 0.04^b^
C10:0	4.83 ± 0.03^a^	6.75 ± 0.01^b^
C12:0	3.74 ± 0.08^a^	4.16 ± 0.01^b^
C14:0	13.76 ± 0.02^a^	14.83 ± 0.07^b^
C16:0	33.14 ± 0.28^a^	31.86 ± 0.02^b^
C17:0	0.75 ± 0.04^a^	0.45 ± 0.03^b^
C18:0	8.27 ± 0.06^a^	7.95 ± 0.06^b^
C20:0	0.29 ± 0.01^a^	0.11 ± 0.01^b^
C22:0	0.16 ± 0.00^a^	0.12 ± 0.02^b^
Σ SFA	70.27 ± 0.62^a^	72.13 ± 0.44^b^
C14:1 n5	1.15 ± 0.00^a^	1.23 ± 0.02^b^
C15:1 n5	0.75 ± 0.01^a^	0.80 ± 0.07^b^
C16:1 n7	1.35 ± 0.06^a^	1.86 ± 0.02^b^
C17:1 n8	0.71 ± 0.00^a^	0.73 ± 0.05^b^
C18:1 n9	15.37 ± 0.12^a^	17.83 ± 0.02^b^
C20:1 n9	0.04 ± 0.05^a^	0.00 ± 0.00^b^
Σ MUFA	19.37 ± 0.23^a^	20.56 ± 0.18^b^
C18:2 n6	1.09 ± 0.03^a^	1.54 ± 0.02^b^
C18:3 n3	0.13 ± 0.01^a^	0.21 ± 0.04^b^
Σ PUFA	1.22 ± 0.04^a^	2.75 ± 0.06^b^
C14:1 t9	0.26 ± 0.02^a^	0.45 ± 0.00^b^
C16:1 t9	0.35 ± 0.04^a^	0.33 ± 0.04^b^
C18:1 t11	0.31 ± 0.02^a^	0.21 ± 0.06^b^
C18:1 t9	0.85 ± 0.04^a^	0.72 ± 0.03^b^
C18:2 t9, t12	0.26 ± 0.02^a^	0.35 ± 0.01^b^
Σ TUFA	2.03 ± 0.14^a^	2.21 ± 0.14^b^
CLA c9t11	0.84 ± 0.03^a^	1.90 ± 0.06^b^
CLA t10c12	0.14 ± 0.01^a^	0.30 ± 0.04^b^
Σ CLA	0.98 ± 0.04^a^	2.20 ± 0.10^b^

*Note*: Means in the identical raw with distinct superscript letters differ *p* < .05.

Abbreviations: SFA, Saturated fatty acid; MUFA, Monounsaturated fatty acid; PUFA, Polyunsaturated fatty acid; TFA, Trans fatty acid; CLA, Conjugated linoleic acid; CIKBO, Iranian butter oil produced from cow's milk; EIKBO, Iranian butter oil produced from ewe's milk.

^a^ Mean of three replicates.

Short‐ and medium‐chain fatty acids, such as caprylic (C8:0) (EIKBO: 2.62, CIKBO: 1.28 (g 100/g)), capric (C10:0) (EIKBO: 6.75, CIKBO: 4.83 (g 100/g)), and lauric acid (C12:0) (EIKBO: 4.16, CIKBO: 3.74 (g 100/g)) were shown to be more abundant in ewe milk than those in cow milk, respectively.

Total saturated fatty acids (g 100/g oil) were found to be 70.27 ± 0.62 and 72.13 ± 0.84 (g 100/g oil) in both CIKBO and EIKBO samples, respectively, which was more than overall unsaturated fatty acids, ranged from CIKBO: 20.59 ± 0.27 to EIKBO: 23.31 ± 0.24 (g 100/g oil). Overall unsaturated fatty acids are divided into total monounsaturated fatty acids (MUFA) and total polyunsaturated fatty acids (PUFA). These findings corroborate prior research on various butter oils derived from cow's milk.

In a study, the saturated fatty acids (SFAs) content of “Fulani butter oil” derived from cow's milk was reported at 53.3 (g 100/g oil), in which the palmitic acid is the predominant fatty acid (30.2 (g 100/g oil)) (Glew et al., 1999). Also, in the other study, the SFAs in butter oil generated from cow and ewe milk obtained 67.06 (g 100/g oil) and 69.10 (g 100/g oil), respectively, and palmitic acid was the predominant fatty acid in both butter oils (Sağdıç et al., 2004). Additionally, the SFAs value of butter oil derived from buffalo milk was obtained at 70.72 (g 100/g oil), and palmitic acid was the major fatty acid (31.89 (g 100/g oil)) (Fatouh et al., 2007). The SFAs content of Tunisian traditional cow milk butter oil was 71.84 (g 100/g oil), and palmitic acid was likewise the predominant fatty acid (21.62 (g 100/g oil)) (Abbas et al., 2021; Samet‐Bali et al., 2009).

The SFAs content of butter oil generated from ewe's milk was obtained at 59.13 (g 100/g oil), with oleic acid being the predominant fatty acid (31.08 (g 100/g oil)) (Özkanlı & Kaya, 2007) which oleic acid value was further than that obtained in this study. The concentration of SFAs in ewe's milk butter oil (72.13 (g 100/g oil)) in this study was much more significant than those in previous reports by Abbas et al. (2021) and Özkanlı & Kaya (2007).

The MUFA concentration in IKBO specimens prepared from cow or ewe cases was 19.37 ± 0.74 (g 100/g butter oil) and 20.56 ± 0.97 (g 100/g butter oil), respectively, with oleic acid being the major fatty acid of the samples' MUFA. The oleic acid values of the CIKBO and EIKBO samples were obtained at 15.37 ± 0.12 (g 100/g butter oil) and 17.83 ± 0.02 (g 100/g butter oil), respectively. Palmitoleic acid was the second most abundant MUFA (CIKBO: 1.35 ± 0.06, EIKBO:1.86 ± 0.02 (g 100/g butter oil)). Previous investigations showed a MUFA value of 20.22 (g 100/g oil) in butter prepared from sheep's milk, which is consistent with the findings of this study (Sağdıç et al., 2004).

Also, the MUFA level of butter oil produced from cow's milk (19.37 (g 100/g butter oil)) was less than previous reports by Dhibi et al. (2013) and Samet‐Bali et al. (2009).

Prior studies showed that the MUFA amount of the total fatty acids in Fulani butter oil was 32 (g 100/g butter oil), which is greater than the value observed in this study (Glew et al., 1999).

The CIKBO and EIKBO samples had PUFAs levels of 1.22 ± 0.12 (g 100/g oil) and 2.75 ± 0.38 (g 100/g oil), respectively, with linoleic acid being the predominant PUFAs. The levels of linoleic and linolenic acids of CIKBO and EIKBO ranged between 1.09 ± 0.03 (g 100/g oil) and 1.54 ± 0.02 (g 100/g oil) and 0.13 ± 0.01 (g 100/g oil) and 0.21 ± 0.04 (g 100/g oil), respectively. These results concurred with prior studies by Sağdıç et al. (2004) and Dhibi et al. (2013).

The PUFAs level of Fulani butter oil was 3.32 (g 100/g oil), which is greater than the current study's results (Glew et al., 1999).

Accordingly, the CIKBO and EIKBO samples had trans unsaturated fatty acids (TUFAs) amounts of 2.03 ± 0.14 (g 100/g butter oil) and 2.21 ± 0.14 (g 100/g butter oil). Trans elaidic acid was determined to be the trans fatty acid with the greatest amount (EIKBO: 0.72 ± 0.03, CIKBO 0.85 ± 0.04 (g 100/g butter oil)) in all datasets. Other trans fatty acids detected in all cases were C14:1 t9, C16:1 t9, C18:1 t11, and C18:2 t9t12.

The total TUFAs levels of the CIKBO and EIKBO samples were significantly greater than those reported by Sağdıç et al. (2004) and Dhibi et al. (2013). In this study, the mean total CLA values of the CIKBO and EIKBO datasets were 0.98 ± 0.04 (g 100/g butter oil) and 2.20 ± 0.10 (g 100/g butter oil), respectively, of total fatty acids. CLA c9, t11 was the predominant CLA isomer in all cases.

Prior investigations revealed that the overall CLA content of sheep milk fat is much greater than that of cow or goat milk fat at 1.08 (g 100/g fat), 1.01(g 100/g fat), and 0.65 (g 100/g fat), respectively. The animal diet, the specific attributes of the milk used to produce butter oil, with particular reference to the species and CLA content of the milk, as well as the processing and manufacturing techniques, have a substantial impact on the quantity of CLA in dairy products, which vary seasonally owing to variabilities in feeding aspects (Chilliard et al., 2003; Prandini et al., 2007; Sindhuja et al., 2020). The most extensive seasonal variations of CLA were seen in sheep milk, which ranged from 1.28 (g 100/g oil) in the summer to 0.54 (g 100/g oil) at the end of the winter (Indu & Jayaprakasha, 2021).

The CLA (C18:2 c9t11) amounts of various Turkish kinds of cheese prepared using conventional techniques ranged between 0.44 and 1.04 (g 100/g oil) (Donmez et al., 2005). The CLA content and the composition of fatty acids of several Turkish dairy products (butter, processed cheese, Kaymak, and cream) were studied, and it discovered that the CLA amounts ranged from 0.97 to 1.05 (g 100/g fat) and that rumenic acid was detected as the predominant CLA isomer in Turkish dairy products (Seçkin et al., 2005). The total CLA content of 12 different French kinds of cheese ranged from 5.3 to 15.80 mg/g of fat (Lavillonnière et al., 1998).

### 3.3 Investigation of triglyceride profile of Kurdish CIKBO and EIKBO, and CRM

Table 4 summarizes the authorized free cholesterol and triglyceride contents of CRM as determined throughout the certification process. Free cholesterol and triglyceride C24–C54 are standardized to 100% and expressed as a mass proportion (percentages). The certified results were derived throughout the approved sets' means, with a confidence interval of 95% around the average and (quadratic addition) the inaccuracy introduced by the reference standard. Hence, the triglycerides for EIKBO are comparable with the TAG of Ewe's fat (Goudjila et al., 2003). The injected material displayed excellent homogeneity and the requisite stability when the triglyceride percentage was determined. The acquired findings and their comparison to CRM indicate that the obtained data are equivalent to CRM.

**TABLE 4:** Triglyceride profile of IKBOs and CRM of cow's butter.

TGs	CIKBO (%)	EIKBO (%)	CRM (%)	TGs	Kurdish butter (%)	EIKBO (%)	CRM
C24	0.09	0.09	0.20	C40	10.1	8.5	10.5
Cholesterol	0.4	0.4	0.40	C42	7.2	7.3	6.8
C26	0.27	0.3	0.31	C44	6.9	7.1	6.8
C28	0.65	0.8	0.70	C46	7.8	7.3	7.3
C30	1.2	1.5	1.2	C48	9.4	9.1	8.8
C32	2.2	2.4	2.6	C50	11.2	13.1	11.1
C34	5.9	5.1	5.7	C52	9.5	11.2	10.3
C36	11.1	8.4	11.3	C54	4.1	5.3	4.5
C38	13.1	9.7	13.5	C56	0.3	0.2	0.2

## 4 CONCLUSION

This research established that IKBO has both nutritional and functional qualities of interest. The physicochemical properties, fatty acid composition, and CLA content of IKBO prepared from the ewe or cow milk were approximately equivalent to those of other butter oils produced historically across the globe. On the other hand, the CLA content of EIKBO was much greater than that of other butter oils reported in other publications. Iranian butter oil was produced using the traditional process employed in the country's northwestern region. The findings indicated that the butter oils have distinct physicochemical properties.

Moreover, the results revealed that the total quantity of free fatty acids and peroxide values in the CIKBO and EIKBO were much more significant than previous reports for butter oils. Myristic, palmitic, stearic, capric, and oleic acids were the most abundant fatty acids in the CIKBO and EIKBO. The butter oil from ewe's milk included the most outstanding levels of CLA and trans elaidic acid, the most prevalent trans fatty acid found in ruminant milk fat. Additional study is required to determine the CLA and trans fatty acids content of IKBOs used in food processing and manufacturing and their health effects.

## FUNDING INFORMATION

This work is not supported with research funding.

## CONFLICT OF INTEREST

Authors declare that there is no conflict of interest.

## Data Availability

Research data are not shared.

## References

[fsn33142-bib-0001] Abbas, H. , El‐Hamid, A. , Badawy, L. , & Salama, M. I. (2021). Vital lipids contents in Buffalo butter oil and its fractions prepared by dry fractionation. Egyptian Journal of Chemistry, 64(2), 4–8.

[fsn33142-bib-0002] Aneja, R. , & Murthi, T. (1990). Conjugated linoleic acid contents of Indian curds and ghee. Indian Journal of Dairy Science, 43(2), 231–238.

[fsn33142-bib-0003] Association of Official Analytical Chemists (AOAC) . (2019). Official methods of analysis (21st ed.). AOAC.

[fsn33142-bib-0004] Batmanglij, N. (1999). A taste of Persia: An introduction to Persian cooking. Mage Publishers.

[fsn33142-bib-0005] Bauman, D. E. , & Griinari, J. M. (2003). Nutritional regulation of milk fat synthesis. Annual Review Nutrition., 23, 203–227. 10.1146/annurev.nutr.23.011702.073408 12626693

[fsn33142-bib-0006] Chilliard, Y. , Ferlay, A. , Rouel, J. , & Lamberet, G. (2003). A review of nutritional and physiological factors affecting goat milk lipid synthesis and lipolysis. Journal of Dairy Science, 86(5), 1751–1770. 12778586 10.3168/jds.S0022-0302(03)73761-8

[fsn33142-bib-0007] Codex Standard . (1999). Milk fat Products. Formerly Codex Stan A‐2‐1973. Adopted in 1973. Codex Stan 280‐1973, Amendment. 2006, 2010, 2018. Codex Stan 280‐1973, Amendment.

[fsn33142-bib-0008] Collomb, M. , Bütikofer, U. , Sieber, R. , Jeangros, B. , & Bosset, J. O. (2002). Correlation between fatty acids in cows' milk fat produced in the lowlands, mountains and highlands of Switzerland and botanical composition of the fodder. International Dairy Journal, 12(8), 661–666.

[fsn33142-bib-0009] Commission Regulation (EC) No 454/95 . (1995). Annex III: Reference method for the detection of foreign fats in milk fat by gas chromatographic analysis of triglycerides. Official Journal, L46, 1.

[fsn33142-bib-0010] Crumb, D. , & Vattem, D. (2011). Conjugated linoleic acid (CLA)‐an overview. International Journal of Applied Research in Natural Products, 4(3), 12–15.

[fsn33142-bib-0011] Dhibi, M. , Mnari, A. , Brahmi, F. , Mechri, B. , Cheraeif, I. , Gazzah, N. , & Hammami, M. (2013). Effect of heat processing on the profiles of trans fatty acids and conjugated linoleic acid in butter oil. African Journal of Biotechnology, 12(21), 3333–3340.

[fsn33142-bib-0012] Donmez, M. , Kemal Seckin, A. , Sagdic, O. , & Simsek, B. (2005). Chemical characteristics, fatty acid compositions, conjugated linoleic acid contents and cholesterol levels of some traditional Turkish cheeses. International Journal of Food Sciences and Nutrition, 56(3), 157–163. 16009630 10.1080/09637480500131137

[fsn33142-bib-0013] Fatouh, A. , Mahran, G. , El‐Ghandour, M. , & Singh, R. (2007). Fractionation of buffalo butter oil by supercritical carbon dioxide. LWT–Food Science and Technology, 40(10), 1687–1693.

[fsn33142-bib-0014] Fındık, O. , & Andiç, S. (2017). Some chemical and microbiological properties of the butter and the butter oil produced from the same raw material. LWT–Food Science and Technology, 86, 233–239.

[fsn33142-bib-0015] Fontecha, J. , Díaz, V. , Fraga, M. J. , & Juárez, M. (1998). Triglyceride analysis by gas chromatography in assessment of authenticity of goat milk fat. Journal of the American Oil Chemists' Society, 75(12), 1893–1896. 10.1007/s11746-998-0347-6

[fsn33142-bib-0016] Glew, R. H. , Okolo, S. N. , Chuang, L. T. , Huang, Y. S. , & VanderJagt, D. (1999). Fatty acid composition of fulani ‘butter oil’ made from cow's milk. Journal of Food Composition and Analysis, 12(3), 235–240.

[fsn33142-bib-0017] Goudjila, H. , Javier, F. M. , Jesús, F. , & Manuela, J. (2003). TAG composition of Ewe's Milk fat. Detection of Foreign Fats. JAOCS, 80(3), 219–222.

[fsn33142-bib-0018] Griinari, J. , Corl, B. , Lacy, S. , Chouinard, P. , Nurmela, K. , & Bauman, D. (2000). Conjugated linoleic acid is synthesized endogenously in lactating dairy cows by Δ9‐desaturase. The Journal of Nutrition, 130(9), 2285–2291. 10958825 10.1093/jn/130.9.2285

[fsn33142-bib-0019] Grundy, S. M. (1997). What is the desirable ratio of saturated, polyunsaturated, and monounsaturated fatty acids in the diet? The American Journal of Clinical Nutrition, 66(4), 988 S–990 S. 10.1093/ajcn/66.4.988S 9322578

[fsn33142-bib-0020] Guler, G. O. , Cakmak, Y. S. , Zengin, G. A. , & Akyildiz, K. (2010). Fatty acid composition and conjugated linoleic acid (CLA) content of some commercial milk in Turkey. Kafkas Üniversitesi Veteriner Fakültesi Dergisi, 16, S37–S40.

[fsn33142-bib-0021] House, R. , Cassady, J. , Eisen, E. , McIntosh, M. , & Odle, J. (2005). Conjugated linoleic acid evokes delipidation through the regulation of genes controlling lipid metabolism in adipose and liver tissue. Obesity Reviews, 6(3), 247–258. 16045640 10.1111/j.1467-789X.2005.00198.x

[fsn33142-bib-0022] Indu, B. , & Jayaprakasha, H. M. (2021). Conjugated linoleic acid‐the natural trans fat: A review. Asian Journal of Dairy and Food Research, 40(4), 351–357.

[fsn33142-bib-0023] INSO 1254 . (2020). Butter oil ‐specifications and test methods. INSO.

[fsn33142-bib-0024] Internationaler, M. (1966). Estimation of the fat content of butter oil. International Standard FIL/IDF 24: 1964. Milchwissenschaft, 21, 139–140.

[fsn33142-bib-0025] ISO 15884 . (2002). Milk fat — Preparation of fatty acid methyl esters [IDF 182:2002]. ISO.

[fsn33142-bib-0026] ISO 17678 . (2019). Milk and milk products — Determination of milk fat purity by gas chromatographic analysis of triglycerides. ISO.

[fsn33142-bib-0027] ISO 19662 . (2018). Milk — Determination of fat content — Acido‐butyrometric (Gerber method) [IDF 238:2018]. ISO.

[fsn33142-bib-0028] ISO 26323 . (2009). Milk products — Determination of the acidification activity of dairy cultures by continuous pH measurement (CpH). ISO.

[fsn33142-bib-0029] ISO 3976 . (2006). Milk fat — Determination of peroxide value [IDF 74:2006]. ISO.

[fsn33142-bib-0030] ISO 660 . (2020). Animal and vegetable fats and oils ‐ Determination of acid value and acidity ‐ Test method. ISO.

[fsn33142-bib-0031] Kay, J. , Weber, W. , Moore, C. , Bauman, D. , Hansen, L. , Chester‐Jones, H. , Crooker, B. , & Baumgard, L. (2005). Effects of week of lactation and genetic selection for milk yield on milk fatty acid composition in Holstein cows. Journal of Dairy Science, 88(11), 3886–3893. 16230694 10.3168/jds.S0022-0302(05)73074-5

[fsn33142-bib-0032] Kelly, M. L. , Berry, J. R. , Dwyer, D. A. , Griinari, J. , Chouinard, P. Y. , Van Amburgh, M. E. , & Bauman, D. E. (1998). Dietary fatty acid sources affect conjugated linoleic acid concentrations in milk from lactating dairy cows. The Journal of Nutrition, 128(5), 881–885. 9566998 10.1093/jn/128.5.881

[fsn33142-bib-0033] Kepler, C. R. , & Tove, S. (1967). Biohydrogenation of unsaturated fatty acids. Journal of Biological Chemistry, 242(24), 5686–5692. 5633396

[fsn33142-bib-0034] Khodamoradi, S. , Fatahnia, F. , Taherpour, K. , Pirani, V. , Rashidi, L. , & Azarfar, A. (2013). Effect of monensin and vitamin E on milk production and composition of lactating dairy cows. Journal of Animal Physiology and Animal Nutrition, 97(4), 666–667. 22533457 10.1111/j.1439-0396.2012.01307.x

[fsn33142-bib-0035] Lavillonnière, F. , Martin, J. , Bougnoux, P. , & Sébédio, J. L. (1998). Analysis of conjugated linoleic acid isomers and content in French cheeses. Journal of the American Oil Chemists' Society, 75(3), 343–352.

[fsn33142-bib-0036] Ledoux, M. , Chardigny, J. M. , Darbois, M. , Soustre, Y. , Sébédio, J. L. , & Laloux, L. (2005). Fatty acid composition of French butters, with special emphasis on conjugated linoleic acid (CLA) isomers. Journal of Food Composition and Analysis, 18(5), 409–425.

[fsn33142-bib-0037] Lokuruka, M. N. I. (2007). Role of fatty acids of milk and dairy products in cardiovascular diseases: A review. African Journal of Food, Agriculture, Nutrition and Development, 7(1), 1–16.

[fsn33142-bib-0038] Mehta, B. M. (2009). Butter, butter oil, and ghee. In Gourmet and health‐promoting specialty oils (pp. 527–559). AOCS Press.

[fsn33142-bib-0039] Nagpal, R. , Behare, P. , Kumar, M. , Mohania, D. , Yadav, M. , Jain, S. , Menon, S. , Parkash, O. , Marotta, F. , Minelli, E. , Henry, C. J. K. , & Yadav, H. (2012). Milk, milk products, and disease free health: An updated overview. Critical Reviews in Food Science and Nutrition, 52, 321–333. 22332596 10.1080/10408398.2010.500231

[fsn33142-bib-0040] Özkanlı, O. , & Kaya, A. (2007). Storage stability of butter oils produced from sheep's non‐pasteurized and pasteurized milk. Food Chemistry, 100(3), 1026–1031.

[fsn33142-bib-0041] Park, Y. , Albright, K. J. , Liu, W. , Storkson, J. M. , Cook, M. E. , & Pariza, M. W. (1997). Effect of conjugated linoleic acid on body composition in mice. Lipids, 32(8), 853–858. 9270977 10.1007/s11745-997-0109-x

[fsn33142-bib-0042] Parodi, P. W. (1994). Conjugated linoleic acid: An anticarcinogenic fatty acid present in milk fat. Australian Journal of Dairy Technology, 49(2), 93–97.

[fsn33142-bib-0043] Parodi, P. W. (1997). Cows' milk fat components as potential anticarcinogenic agents. The Journal of Nutrition, 127(6), 1055–1060. 9187617 10.1093/jn/127.6.1055

[fsn33142-bib-0044] Parodi, P. W. (2003). Conjugated linoleic acid in food. Advances in conjugated linoleic acid research, 2, 101–122.

[fsn33142-bib-0045] Prandini, A. , Sigolo, S. , Tansini, G. , Brogna, N. , & Piva, G. (2007). Different level of conjugated linoleic acid (CLA) in dairy products from Italy. Journal of Food Composition and Analysis, 20(6), 472–479.

[fsn33142-bib-0046] Sağdıç, O. , Dönmez, M. , & Demirci, M. (2004). Comparison of characteristics and fatty acid profiles of traditional Turkish yayik butters produced from goats', ewes' or cows' milk. Food Control, 15(6), 485–490.

[fsn33142-bib-0047] Samet‐Bali, O. , Ayadi, M. , & Attia, H. (2009). Traditional Tunisian butter: Physicochemical and microbial characteristics and storage stability of the oil fraction. LWT–Food Science and Technology, 42(4), 899–905.

[fsn33142-bib-0048] Seçkin, A. K. , Gursoy, O. , Kinik, O. , & Akbulut, N. (2005). Conjugated linoleic acid (CLA) concentration, fatty acid composition and cholesterol content of some Turkish dairy products. LWT–Food Science and Technology, 38(8), 909–915.

[fsn33142-bib-0049] Şenel, E. , Atamer, M. , & Öztekin, F. Ş. (2011). The oxidative and lipolytic stability of Yayık butter produced from different species of mammal's milk (cow, sheep, and goat) yoghurt. Food Chemistry, 127(1), 333–339.

[fsn33142-bib-0050] Sindhuja, S. , Prakruthi, M. , Manasa, R. , & Shivananjappa, M. (2020). Health benefits of ghee (clarified butter)‐A review from ayurvedic perspective. IP Journal of Nutrition, Metabolism and Health Science, 3(3), 64–72.

[fsn33142-bib-0051] Uzatici, A. , & Yayintaş, O. T. (2017). Determination of the quality of raw milk from black and white cows from Biga (Canakkale, Turkey). Journal of Scientific Perspectives, 1, 29–42.

